# The multi-protective role of dietary betaine in largemouth bass (*Micropterus salmoides*): coordinating antioxidant, inflammatory, and metabolic homeostasis under high-fat diet stress

**DOI:** 10.3389/fphys.2025.1742669

**Published:** 2025-12-10

**Authors:** Yi-Jia Liu, Jian Guo, Chang-Da Li, Si-Yin Han, Lu Cai

**Affiliations:** 1 Food and Pharmacy College, Zhejiang Ocean University, Zhoushan, China; 2 Wenzhou Dongtou District Marine and Fisheries Development Research Center, Wenzhou, China; 3 College of Food Science and Engineering, Henan University of Technology, Zhengzhou, China; 4 Zhoushan Sailaite Marine Technology Co., Ltd., Zhoushan, China; 5 College of Medicine, Zhejiang Ocean University, Zhoushan, China

**Keywords:** betaine, *Micropterus salmoides*, high-fat diet, antioxidant capacity, lipid metabolism, inflammatory response

## Abstract

**Introduction:**

Intensive aquaculture frequently utilizes high-fat diets (HF) as a cost-effective strategy, yet this practice often induces hepatic steatosis, oxidative stress, and chronic inflammation in carnivorous fish. Betaine, a natural methyl donor, has shown potential as a functional feed additive, but its comprehensive protective mechanisms under HF stress remain to be fully elucidated.

**Methods:**

Juvenile largemouth bass (Micropterus salmoides) were fed one of four isonitrogenous diets for 8 weeks: a normal-fat control (Control), a high-fat diet (HF), and two high-fat diets supplemented with 0.5% (HFB0.5) or 1.0% (HFB1) betaine. Growth performance, digestive enzyme activities, serum biochemical parameters, hepatic antioxidant capacity, and the expression of genes related to antioxidant defense, lipid metabolism, and inflammation were analyzed.

**Results:**

The HF group exhibited significantly impaired growth, digestive function, and antioxidant capacity, along with elevated lipid peroxidation, dyslipidemia, and pro-inflammatory cytokine expression. Betaine supplementation restored growth performance and feed efficiency to control levels, ameliorated digestive enzyme activities (particularly enhancing lipase), and activated the hepatic Nrf2-Keap1 pathway, upregulating antioxidant genes (nrf2, sod1, cat, gpx, ho-1, gr) and enhancing enzyme activities. Betaine also improved serum lipid profiles, upregulated genes related to fatty acid oxidation (pparα, cpt-1) and lipolysis (lpl, hsl), suppressed lipogenic genes (srebp-1, fas), and rebalanced inflammatory cytokines by reducing tnf-α and il-1β while increasing tgf-β1 and il-10.

**Discussion:**

Dietary betaine effectively counteracts HF-induced metabolic stress in M. salmoides through coordinated multi-pathway regulation. It enhances antioxidant defense, reprograms hepatic lipid metabolism toward catabolism, and restores inflammatory homeostasis. These findings underscore betaine’s role as a multi-functional feed additive capable of mitigating HF-related metabolic disorders and promoting overall health in carnivorous fish aquaculture.

## Introduction

The intensive aquaculture industry frequently employs high-fat diets to promote growth performance and improve feed efficiency, as lipids serve as a cost-effective energy source that can spare dietary protein for growth (Li et al., 2012; Xie et al., 2021). However, prolonged feeding of high-fat diets often leads to adverse metabolic consequences in fish, including hepatic steatosis, dyslipidemia, oxidative stress, and chronic inflammation, which collectively impair health and productivity (Cao et al., 2019; Dai et al., 2018). Carnivorous fish species such as largemouth bass (*Micropterus salmoides*) are particularly susceptible to these metabolic disorders due to their limited capacity for dietary lipid metabolism, resulting in reduced growth and compromised immune competence (Jin et al., 2021; Liang et al., 2023). The high-fat diet used in the present study was formulated based on recent research on *M. salmoides*, which indicates that lipid levels around 15%–18% can induce significant metabolic stress and serve as a robust model for studying nutritional interventions, while being representative of practical feed formulations aiming for protein sparing (Zhao et al., 2024; Zhang et al., 2025).

Betaine, a natural methyl donor and osmolyte, has gained increasing attention as a functional feed additive in aquafeeds to counteract the negative effects of high-fat diets (Jin et al., 2021; Adjoumani et al., 2017). The efficacy of betaine as a dietary supplement to boost growth performance and enhance the appeal of feed has been documented in numerous fish species (Li et al., 2024; Mohseni et al., 2021). Beyond its growth-promoting effects, betaine shows a remarkable capacity to regulate lipid metabolism. The anti-lipidemic effect of betaine is evidenced in *Acanthopagrus schlegelii* and *Carassius auratus gibelio*, where it appears to alleviate hepatic lipid deposition by orchestrating the activity of key transcriptional regulators and enzymes that govern fatty acid synthesis and oxidation, thereby restoring metabolic homeostasis (Jin et al., 2021; Dong et al., 2018). Furthermore, its anti-inflammatory and antioxidant properties have been reported in models of metabolic stress, where betaine supplementation was found to suppress the expression of pro-inflammatory cytokines and enhance the activities of antioxidant enzymes (Sun et al., 2020; He et al., 2024).

Notwithstanding the evidence, the precise mechanisms by which betaine exerts its hepatoprotective effects in the context of high-fat feeding in fish remain to be fully characterized. Existing research has often focused on isolated aspects, such as growth or lipid metabolism, leaving a gap in our knowledge regarding its simultaneous impact on the interconnected networks of antioxidant defense and inflammatory response. Specifically, the role of betaine in activating core cytoprotective signaling pathways, such as the Nrf2-Keap1 system, which governs the expression of a battery of antioxidant genes, is poorly characterized in this context. Further clarification is required to understand the mechanisms by which betaine balances pro-inflammatory and anti-inflammatory signals and maintains inflammatory homeostasis in fish receiving a high-fat diet.

The primary objective of this study was to comprehensively assess the efficacy of dietary betaine in mitigating the adverse effects of a high-fat diet in *M. salmoides* and to uncover the associated molecular mechanisms. The investigation focused on elucidating whether betaine exerts its beneficial roles through multi-pathway regulation, including enhancing cellular antioxidant capacity, restoring inflammatory homeostasis, and reprogramming hepatic lipid metabolism toward catabolism. The study design incorporated a multifaceted assessment of growth metrics, digestive enzyme function, serum biochemical profiles, and transcriptional regulation of key genes involved in antioxidant defense, inflammatory response, and lipid metabolism. The findings of this study are expected to clarify the synergistic mechanisms underlying the efficacy of betaine and provide a solid scientific foundation for its application as a strategic nutraceutical in high-energy feeds for carnivorous fish.

## Materials and methods

### Diet preparation and experimental design

Betaine (purity ≥98%) was sourced from Aladdin Biochemical Technology Co., Ltd. (Shanghai, China). Four isonitrogenous experimental diets were formulated: a normal-fat control diet (Control), a high-fat diet (HF), and two high-fat diets supplemented with 0.5% or 1.0% betaine, designated as HFB0.5 and HFB1, respectively. Table 1 outlines the specific formulation and proximate composition of the diets. All dry ingredients were first ground to a fine consistency, thoroughly blended, and then processed into 2.5 mm pellets using a twin-screw extruder at 90 °C. The resulting pellets were air-dried to a stable moisture level and stored at −20 °C until use. The proximate composition of the finished diets was determined following the official AOAC (2012) methods.

**TABLE 1:** Formulation and proximate composition of experimental diets (% dry matter).

Ingredients	Control	HF	HFB0.5	HFB1
Fish meal	35	35	35	35
Soybean meal	16	16	16	16
Wheat gluten	7	7	7	7
Krill meal	2	2	2	2
Soy protein isolate	8.5	8.5	8.5	8.5
Wheat flour	12.3	12.3	12.3	12.3
Fish oil	3	3	3	3
Soybean oil	3	9	9	9
Soybean lecithin	1	1	1	1
Vitamin premix^a^	1	1	1	1
Mineral premix^b^	1	1	1	1
Choline chloride (50%)	0.5	0.5	0.5	0.5
Vitamin C	0.2	0.2	0.2	0.2
Sodium alginate	1	1	1	1
Monocalcium phosphate	1	1	1	1
Microcrystalline cellulose	7.5	1.5	1	0.5
Betaine	0	0	0.5	1
Total	100	100	100	100
Proximate composition				
Dry matter	90.15	90.13	90.24	90.31
Crude protein	42.97	43.11	43.07	42.94
Crude lipid	11.12	17.21	17.14	17.18
Moisture	9.37	9.07	9.01	9.23

^a^ Vitamin premix provides the following per kg of diet: vitamin B1, 30 mg; vitamin B2, 60 mg; vitamin B6, 20 mg; nicotinic acid, 200 mg; calcium pantothenate, 100 mg; inositol, 100 mg; biotin, 2.5 mg; folic acid, 10 mg; vitamin B12, 0.1 mg; vitamin K3, 40 mg; vitamin A, 10,000 IU; vitamin D3, 2,000 IU; vitamin E, 160 IU.

^b^ Mineral premix provides the following per kg of diet: MgSO4·7H2O, 1,090 mg; KH_2_PO_4_, 932 mg; NaH_2_PO_4_·2H_2_O, 432 mg; FeC_6_H_5_O_7_·5H2O, 181 mg; ZnCl_2_, 80 mg; CuSO_4_·5H_2_O, 63 mg; AlCl3·6H_2_O, 51 mg; MnSO_4_·H_2_O, 31 mg; KI, 28 mg; CoCl_2_·6H_2_O, 6 mg; Na_2_SeO_3_·H_2_O, 0.8 mg.

### Fish and feeding management

The experiment utilized juvenile *M. salmoides* sourced from a commercial farm in Huzhou, Zhejiang Province, China. Following a 2-week acclimation, 480 juveniles (initial weight: 9.67 ± 0.07 g) were randomly distributed among 16 tanks (30 fish per tank), with four tanks assigned to each of the four experimental diets. For 8 weeks, fish were fed to apparent satiation two times per day. Water quality was rigorously controlled, maintaining a temperature of 27.5 °C ± 1.0 °C, dissolved oxygen above 6.5 mg/L, and total ammonia nitrogen below 0.1 mg/L. The Institutional Animal Care and Use Committee of Zhejiang Ocean University approved all experimental protocols.

### Sample collection and tissue processing

Following the completion of the 8-week feeding trial, the fish were fasted for 24 h before sample collection. All fish were anesthetized and subsequently euthanized using eugenol at a concentration of 100 mg/L. Final biomass and survival rate were determined per tank for subsequent growth performance evaluation. Blood was drawn from the caudal vein of six randomly selected fish per replicate tank. Following collection, samples were kept at 4 °C to coagulate and then centrifuged (5,000 × g, 10 min, 4 °C). The resulting serum supernatant was aliquoted and stored at −80 °C for subsequent biochemical analysis. An additional six fish per tank were euthanized for tissue sampling. The liver and intestine were excised, immediately snap-frozen in liquid nitrogen, and stored at −80 °C for future analysis. Hepatic samples were reserved for antioxidant enzyme activity determination and gene expression analysis, while intestinal tissues were allocated for digestive enzyme activity assessment.

### Analysis of digestive and antioxidant enzymes

To prepare tissue supernatants for biochemical analysis, liver and intestine samples were processed into homogenates using nine volumes of ice-cold phosphate buffer (pH 7.4) and a mechanical homogenizer. The homogenates were then subjected to centrifugation (3,000 × g, 20 min, 4 °C) to obtain clear supernatants. A comprehensive evaluation of the hepatic antioxidant status was conducted, encompassing the activities of key antioxidant enzymes (superoxide dismutase, SOD; catalase, CAT; glutathione peroxidase, GPX), the total antioxidant capacity (T-AOC), and the concentration of malondialdehyde (MDA). Concurrently, digestive enzyme activities (α-amylase, trypsin, and lipase) were determined in intestinal supernatants to evaluate digestive function. Specific commercial kits from the Nanjing Jiancheng Bioengineering Institute (Nanjing, China) were employed for all enzymatic analyses, strictly in accordance with the manufacturer’s protocols. Absorbance values were recorded with a spectrophotometer, and enzyme activities were calculated according to established methodologies described in the kit instructions. The specific measurement methods for all the aforementioned biochemical indices were performed following the procedures described by Li et al. (2021).

### Serum biochemical analysis

The following serum biochemical parameters were quantified using commercial assay kits (Nanjing Jiancheng Bioengineering Institute, China): total cholesterol (TC), triglycerides (TG), low-density lipoprotein cholesterol (LDL-C), and high-density lipoprotein cholesterol (HDL-C). All determinations were performed following the manufacturer’s guidelines, with absorbance readings obtained through spectrophotometric methods. The analytical procedures were carried out using enzymatic colorimetric methods as specified in the respective kit protocols.

### RNA extraction and quantitative real-time PCR analysis

Total RNA isolation from liver tissues was performed with TRIzol reagent (TaKaRa, Dalian, China) in accordance with the manufacturer’s instructions. RNA quality and concentration were verified by spectrophotometry. Using the PrimeScript RT reagent Kit (TaKaRa), 1 μg of total RNA was reverse-transcribed into cDNA after genomic DNA elimination. This cDNA served as template for real-time PCR amplification performed with SYBR Green Premix on a LightCycler 480 II detection system (Roche, Switzerland). Amplification was carried out through the following protocol: 60 s at 95 °C for initial denaturation, then 40 cycles of three-step amplification (5 s at 95 °C, 15 s at 60 °C, and 20 s at 72 °C). Relative transcript abundances were determined via the 2^−ΔΔCT^ algorithm following normalization to the endogenous reference gene *ef-1a*. Corresponding primer sequences appear in Table 2.

**TABLE 2:** Primer sequences used for real-time quantitative PCR analysis.

Gene name	Primer sequence (5′–3′)	Sources/Genbank accession no.
*nrf2*	F-CTCTGTTCCCAGTATGGCCC R-GAAGGGAGGCTTGTTTGGGA	XM_038720536.1
*gr*	F-CTGAAGTTCCAGGGGCAAGT R-TGCAATATAACCCGCACCGA	XM_038700350.1
*ho-1*	F-ATGGTGATTGTCCCCTCAGC R-CTCAGCCCGAGGGATTTCTG	XM_038694281.1
*sod1*	F- TGGCAAGAACAAGAACCACA R- CCTCTGATTTCTCCTGTCACC	XM_038708943.1
*cat*	F- ATCCCTGTGGGCAAAATGGT R- CGGTGACGATGTGTGTCTGG	XM_038704976.1
*gpx*	F- GTATGTCCGTCCAGGGAATGG R- TCCTACAGACGGGACTCCAAA	XM_038697220.1
*il-1β*	F- CGTGACTGACAGCAAAAAGAGG R- GATGCCCAGAGCCACAGTTC	Yu et al., 2018
*tnf-α*	F-CTTCGTCTACAGCCAGGCATCG R-TTTGGCACACCGACCTCACC	XM_038710731.1
*tgf-β1*	F- GCTCAAAGAGAGCGAGGATG R- TCCTCTACCATTCGCAATCC	Yu et al., 2018
*il-10*	F- CGGCACAGAAATCCCAGAGC R- CAGCAGGCTCACAAAATAAACATCT	XM_038696252.1
*pparα*	F- CCACCGCAATGGTCGATATG R-TGCTGTTGATGGACTGGGAAA	XM_038705497.1
*cpt-1*	F- AGTCGTGTCCCAGGTGTAGA R- CCGCCCGTCATAAAACATC	XM_038705335.1
*lpl*	F- GTCCAAAGATGACGCCCAG R- TTCTGTATGTCGCAGCCCG	MF322778.1
*hsl*	F- TACATCACTGCCAACCGTCG R- GCCATAGAAGCACCCCTTGT	XM_038710965.1
*fas*	F- ACACAATGGGCAGGTATGGG R- GAGTAGGCGAGACACAACCA	XM_038735140.1
*srebp-1*	F- ATGGGGATTGGGCTATTCGC R- GGCTGGGGCTTTTCTCTTCA	XM_038699585.1
*ef-1α*	F- GGCTGGTATTTCCAAGAACG R- GTCTCCAGCATGTTGTCTCC	KT827794.1

Abbreviations: nrf2, nuclear factor erythroid 2-related factor 2; gr, glutathione reductase; ho-1, heme oxygenase 1; sod1, superoxide dismutase 1; cat, catalase; gpx, glutathione peroxidase; il-1β, interleukin 1 beta; tnf-α, tumor necrosis factor alpha; tgf-β1, transforming growth factor beta 1; il-10, interleukin 10; pparα, peroxisome proliferatoractivated receptor alpha; cpt-1, carnitine palmitoyltransferase 1; lpl, lipoprotein lipase; hsl, hormone-sensitive lipase; fas, fatty acid synthase; srebp-1, sterol regulatory element binding transcription factor 1; ef-1a, elongation factor 1 alpha.

### Statistical methods

All values are reported as mean ± standard deviation (SD). Using SPSS 20.0 (SPSS Inc., United States), datasets were first assessed for normality with the Kolmogorov-Smirnov test and for variance homogeneity with Levene’s test. Intergroup differences were then analyzed by one-way ANOVA with Tukey’s multiple comparison test, considering P-values below 0.05 as statistically significant.

## Results

### Growth performance and feed efficiency

The effects of dietary treatments on the growth performance and feed utilization of juvenile *M. salmoides* are presented in Table 3. The growth performance of the HF group was adversely affected, as evidenced by significantly impaired final body weight (FBW), weight gain rate (WGR), specific growth ratio (SGR), and feed efficiency (FE) compared to both the Control and the betaine-supplemented groups (*P* < 0.05), indicating this effect was mitigated by betaine supplementation. Notably, the HFB0.5 and HFB1 groups showed growth and feed utilization metrics (FBW, WGR, SGR, FE) with no significant differences from the Control (*P* > 0.05), indicating that betaine fully compensated for the negative effects of the high-fat diet. No significant differences were detected in survival rate (SR) across all experimental diets (*P* > 0.05).

**TABLE 3:** Growth performance and feed efficiency of juvenile *Micropterus salmoides* fed the experimental diets.

Items	Control	HF	HFB0.5	HFB1
IBW (g)	9.63 ± 0.09	9.65 ± 0.07	9.68 ± 0.09	9.71 ± 0.04
FBW (g)	61.47 ± 1.61^a^	55.52 ± 2.33^b^	61.25 ± 2.92^a^	60.59 ± 2.03^a^
WGR (%)	506.01 ± 8.76^a^	441.63 ± 19.60^b^	501.20 ± 37.72^a^	487.44 ± 30.19^a^
SGR (%/d)	3.31 ± 0.04^a^	3.12 ± 0.08^b^	3.29 ± 0.10^a^	3.27 ± 0.06^a^
FE	1.14 ± 0.02^a^	1.01 ± 0.04^b^	1.13 ± 0.04^a^	1.11 ± 0.04^a^
SR (%)	95.00 ± 1.92	94.17 ± 1.67	95.00 ± 1.92	94.17 ± 1.67

Data are presented as mean ± SD (n = 4). Different superscript letters within a row indicate significant differences (*P* < 0.05).

Weight gain rate (WGR, %) = 100 × (final individual weight − initial individual weight)/initial individual weight.

Specific growth ratio (SGR, % day^-1^) = 100 × (Ln (final individual weight) - Ln (initial individual weight))/number of feeding days.

Feed efficiency (FE) = (final body weight (g) - initial body weight (g))/feed intake (g, dry weight).

Survival rate (SR, %) = 100 × (final number of fish)/(initial number of fish).

### Digestive enzyme activities

The activities of key digestive enzymes in the mid-gut were significantly altered by the experimental diets (Table 4). The HF group exhibited a distinct impairment in carbohydrate and protein digestion, as evidenced by significantly reduced activities of α-amylase and trypsin compared to the Control group (*P* < 0.05). Notably, supplementation with betaine (HFB0.5 and HFB1) completely ameliorated these negative effects. Betaine supplementation restored α-amylase and trypsin activities in the HFB groups to match those in the Control group (*P* > 0.05), demonstrating its efficacy in protecting digestive function from the challenges of a high-fat diet. A contrasting pattern was observed for lipase, the activity of which showed a marked elevation in the HF group relative to the Control (*P* < 0.05). This elevated activity was further increased in the HFB0.5 and HFB1 groups, with both betaine-supplemented groups showing significantly higher lipase activity than both the HF and the Control groups (*P* < 0.05). This suggests that betaine supplementation not only counteracted the impairment of carbohydrate and protein digestion but also actively promoted lipid digestive capacity.

**TABLE 4:** Digestive enzyme activities in the mid-gut of juvenile *Micropterus salmoides* fed the experimental diets.

Items	Control	HF	HFB0.5	HFB1
α-Amylase (U/mg protein)	3.71 ± 0.17^a^	2.39 ± 0.24^b^	3.64 ± 0.30^a^	3.71 ± 0.24^a^
Lipase (U/g protein)	34.35 ± 3.41^c^	52.99 ± 4.39^b^	72.18 ± 4.14^a^	71.73 ± 2.53^a^
Trypsin (U/mg protein)	261.52 ± 5.15^b^	187.40 ± 6.28^c^	282.54 ± 3.34^a^	286.28 ± 11.99^a^

Data are presented as mean ± SD (n = 4). Different superscript letters within a row indicate significant differences (*P* < 0.05).

### Antioxidant enzyme activities

Hepatic antioxidant status varied significantly in response to the different dietary treatments, as detailed in Table 5. A marked suppression of antioxidant capacity was observed in the HF group, with activities of SOD, GPX, and CAT being significantly lower relative to the Control (*P* < 0.05). This impaired antioxidant defense was accompanied by a significantly higher level of MDA in the HF group relative to the Control (*P* < 0.05), indicating elevated lipid peroxidation and oxidative stress. Dietary betaine supplementation effectively mitigated these adverse effects. SOD and CAT activities in the betaine-supplemented groups (HFB0.5, HFB1) were restored to control levels (*P* > 0.05) and were markedly higher than those in the HF group (*P* < 0.05). Unlike the restoration pattern seen for other enzymes, betaine supplementation significantly boosted GPX activity beyond even the Control level (*P* < 0.05). Concurrently, MDA content in these groups was effectively lowered to that of the Control, representing a significant reduction from the HF group (*P* < 0.05).

**TABLE 5:** Hepatic antioxidant enzyme activities and oxidative status of juvenile *Micropterus salmoides* fed the experimental diets.

Items	Control	HF	HFB0.5	HFB1
SOD (U/mg protein)	81.08 ± 6.35^a^	62.94 ± 5.98^b^	89.06 ± 7.38^a^	89.78 ± 6.33^a^
GPX (U/mg protein)	94.86 ± 5.83^b^	81.72 ± 6.31^c^	114.62 ± 9.11^a^	112.78 ± 11.49^a^
MDA (nmol/mg protein)	1.06 ± 0.10^b^	1.56 ± 0.09^a^	1.04 ± 0.11^b^	1.04 ± 0.13^b^
CAT (nmol/mg protein)	24.70 ± 2.75^a^	18.08 ± 1.62^b^	23.70 ± 3.25^a^	24.23 ± 3.48^a^

Data are presented as mean ± SD (n = 4). Different superscript letters within a row indicate significant differences (*P* < 0.05).

### Serum biochemical parameters

The serum biochemical parameters of juvenile *M. salmoides* were significantly influenced by the dietary treatments (Table 6). Compared to the Control group, the HF group demonstrated significantly higher concentrations of TG, TC, and LDL-C, alongside a significantly lower concentration of HDL-C (*P* < 0.05). Dietary betaine supplementation effectively reversed these alterations induced by the high-fat diet. The HFB0.5 and HFB1 groups showed a significant reduction in serum levels of TG, TC, and LDL-C relative to the HF group (*P* < 0.05). Betaine exerted a pronounced lipid-lowering effect, as evidenced by TG concentrations in supplemented groups falling significantly below even the Control level (*P* < 0.05). In contrast, TC levels displayed a partial response, remaining elevated relative to the Control yet significantly suppressed compared to the HF group (*P* < 0.05). A complete reversal was observed for HDL-C and LDL-C, both of which were restored to levels statistically indistinguishable from the Control (*P* > 0.05).

**TABLE 6:** Serum biochemical parameters of juvenile *Micropterus salmoides* fed the experimental diets.

Items	Control	HF	HFB0.5	HFB1
TG	3.17 ± 0.10^b^	5.53 ± 0.09^a^	2.50 ± 0.09^c^	2.45 ± 0.07^c^
TC	12.94 ± 0.14^c^	17.32 ± 0.18^a^	14.43 ± 0.13^b^	14.38 ± 0.10^b^
LDL-C	3.72 ± 0.16^b^	5.69 ± 0.18^a^	3.88 ± 0.47^b^	3.79 ± 0.53^b^
HDL-C	8.58 ± 0.25^a^	6.69 ± 0.14^b^	8.75 ± 0.19^a^	8.64 ± 0.24^a^

Data are presented as mean ± SD (n = 4). Different superscript letters within a row indicate significant differences (*P* < 0.05). TG, triglyceride (mmol L^-1^); TC, total cholesterol (mmol L^-1^); LDL-C, low-density lipoprotein cholesterol (mmol L^-1^); HDL-C, high-density lipoprotein cholesterol (mmol L^-1^).

### Hepatic expression of antioxidant-related genes

**FIGURE 1:**
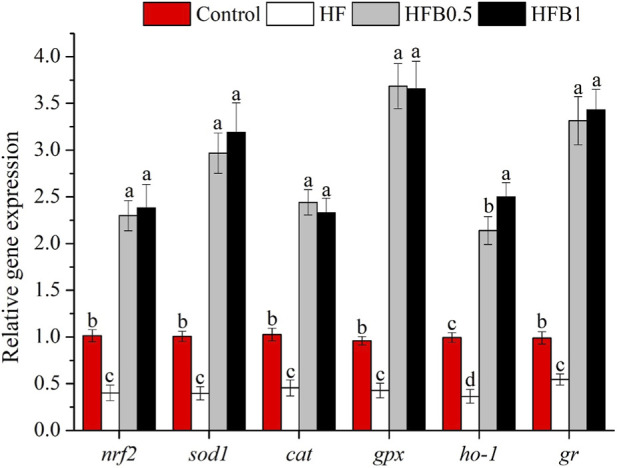
Relative hepatic mRNA expression levels of antioxidant-related genes in juvenile *Micropterus salmoides* fed experimental diets. Data are shown as mean ± SD (n = 4). Different lowercase letters above bars indicate significant differences among groups (*P* < 0.05).

As illustrated in Figure 1, the HF diet significantly downregulated the mRNA expression of antioxidant-related genes, including nuclear factor erythroid 2-related factor 2 (*nrf2*), superoxide dismutase 1 (*sod1*), catalase (*cat*), glutathione peroxidase (*gpx*), heme oxygenase 1 (*ho-1*), and glutathione reductase (*gr*), compared to the Control group (*P* < 0.05). Dietary betaine supplementation markedly upregulated their expression. The transcript levels of all these genes in the HFB0.5 and HFB1 groups were significantly higher than those in both the HF group and the Control group (*P* < 0.05), indicating a potent induction of the antioxidant defense system by betaine.

### Hepatic expression of lipid metabolism-related genes

**FIGURE 2:**
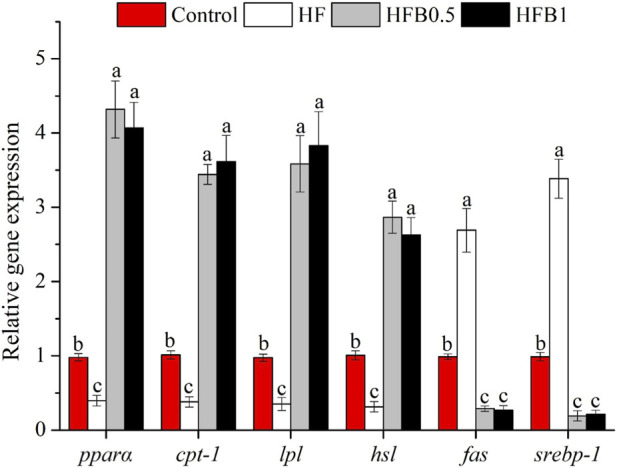
Relative hepatic mRNA expression levels of lipid metabolism-related genes in juvenile *Micropterus salmoides* fed experimental diets. Data are shown as mean ± SD (n = 4). Different lowercase letters above bars indicate significant differences among groups (*P* < 0.05).

The expression profiles of genes involved in hepatic lipid metabolism are summarized in Figure 2. The HF diet promoted a transcriptional profile favoring lipid accumulation, characterized by the significant upregulation of the lipogenic genes fatty acid synthase (*fas*) and sterol regulatory element binding transcription factor 1 (*srebp-1*), while concurrently suppressing genes critical for fatty acid oxidation (peroxisome proliferator-activated receptor alpha (*pparα*), carnitine palmitoyltransferase 1 (*cpt-1*)) and lipolysis (lipoprotein lipase (*lpl*), hormone-sensitive lipase (*hsl*)) compared to the Control group (*P* < 0.05). Dietary betaine supplementation effectively reversed these HF-induced alterations. The HFB0.5 and HFB1 groups showed a significant upregulation in the mRNA abundance of *pparα*, *cpt-1*, *lpl*, and *hsl* relative to both the Control and HF groups (*P* < 0.05). Conversely, the expression of *fas* and *srebp-1* in betaine-supplemented groups was markedly suppressed relative to both the Control and HF groups (*P* < 0.05). These findings demonstrate that betaine supplementation not only enhanced fatty acid oxidation and lipolysis but also concurrently inhibited lipogenic pathways, thereby promoting a metabolic shift toward reduced lipid accumulation.

### Hepatic expression of inflammation-related genes

**FIGURE 3:**
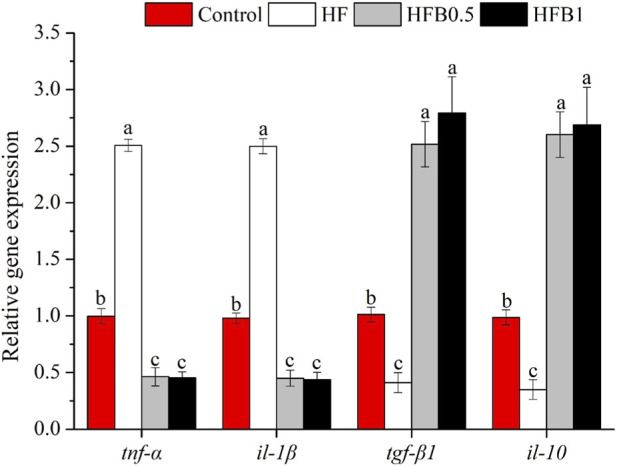
Relative hepatic mRNA expression levels of inflammation-related genes in juvenile *Micropterus salmoides* fed experimental diets. Data are shown as mean ± SD (n = 4). Different lowercase letters above bars indicate significant differences among groups (*P* < 0.05).

The expression profiles of inflammation-related genes are presented in Figure 3. Fish fed the high-fat diet exhibited a marked pro-inflammatory state, as indicated by a significant increase in the transcription of tumor necrosis factor alpha (*tnf-α*) and interleukin 1 beta (*il-1β*) alongside a decrease in transforming growth factor beta 1 (*tgf-β1*) and interleukin 10 (*il-10*) relative to the Control (*P* < 0.05). Dietary betaine supplementation effectively reversed this inflammatory dysregulation. Specifically, the betaine-treated groups (HFB0.5 and HFB1) showed a notable suppression of pro-inflammatory gene expression, with *tnf-α* and *il-1β* transcript levels falling below those of both the Control and HF groups (*P* < 0.05). Conversely, the mRNA abundance of anti-inflammatory mediators *tgf-β1* and *il-10* was significantly elevated in these groups compared to the Control and HF groups (*P* < 0.05). Collectively, these findings indicate that betaine not only attenuated the pro-inflammatory cascade but also actively potentiated the anti-inflammatory response in the liver of juvenile *M. salmoides*.

## Discussion

The restoration of growth performance in *M*. *salmoides* through betaine supplementation highlights its potential application value as a functional additive in high-fat diet formulations. Impaired growth under high-fat dietary stress has been commonly observed in carnivorous fish species, largely due to metabolic inefficiency caused by lipid overload (Guo et al., 2025; Xia et al., 2024). The ability of betaine to fully restore growth performance highlights its effectiveness in counteracting these adverse effects. A key mechanism underlying this improvement appears to be the enhancement of digestive function and feed utilization efficiency. The compromised activities of key digestive enzymes under high-fat feeding conditions likely contribute to reduced nutrient availability, ultimately limiting growth potential (Dawood et al., 2025; Zhao et al., 2023a). Betaine-mediated enhancement of digestive enzyme activities likely contributes to the restoration of growth performance in fish fed high-lipid diets. Betaine serves as a methyl donor in protein and energy metabolism, and previous studies indicate that it can both counteract enzyme inhibition and enhance digestive enzyme activity, thereby improving feed utilization efficiency (Mohseni et al., 2021; Ghosh et al., 2019). This enzymatic optimization, particularly the pronounced stimulation of lipase activity, facilitates more efficient breakdown and absorption of dietary lipids, thereby supporting better feed conversion and growth outcomes.

The antioxidant defense system is essential for maintaining cellular integrity and metabolic homeostasis in fish, particularly under nutritional challenges. The transcription factor Nrf2, through its regulation of the Nrf2-Keap1 pathway, serves as a primary coordinator of cellular antioxidant responses, controlling the expression of various detoxification and antioxidant enzymes (Zhao et al., 2023a). HF diet frequently induces oxidative stress by disrupting this regulatory system, as demonstrated in the current study where lipid-rich feeding impaired antioxidant function. Previous investigations in multiple fish species have documented similar oxidative damage resulting from HF diets (Guo et al., 2025; Dawood et al., 2025; Zhao et al., 2023b). The present study reveals that dietary betaine supplementation effectively activates the Nrf2-mediated antioxidant pathway through coordinated enhancement of both gene expression and enzymatic activities. The comprehensive enhancement of antioxidant capacity observed in this study can be plausibly attributed to the well-documented function of betaine as both an osmolyte and methyl donor in cellular protection. Betaine is known to accumulate in cells under stress to stabilize macromolecular structures and maintain enzymatic activity (Buonaiuto et al., 2025; Khan et al., 2020). This osmoprotective effect likely contributes to the stabilization of the Nrf2 signaling machinery and the observed increase in antioxidant enzyme activities. Furthermore, the methyl-donor property of betaine supports the synthesis of phosphatidylcholine, which is essential for maintaining membrane integrity under oxidative assault and for efficient very-low-density lipoprotein (VLDL) export of lipids from the liver, indirectly reducing oxidative stress (Buonaiuto et al., 2025). Therefore, the potent activation of the Nrf2 pathway by betaine is likely a downstream consequence of its primary role in mitigating broader cellular metabolic stress, thereby creating a cellular environment conducive to robust antioxidant gene expression and function. Earlier studies in aquatic species primarily emphasized the growth-promoting effects of betaine, with limited exploration of its transcriptional regulation of antioxidant defenses (Jin et al., 2021; Mohseni et al., 2021; Luo et al., 2011). The current findings provide evidence that betaine functions as a nutritional modulator that enhances cellular resilience through integrated upregulation of both transcriptional and functional components of the antioxidant system.

Serum biochemical parameters serve as crucial clinical indicators for evaluating systemic metabolic health in fish, reflecting the integrity of multiple physiological processes (Zhao et al., 2020; Fazio, 2019). The dyslipidemic pattern observed in the HF group, characterized by elevated TG, TC, and LDL-C together with reduced HDL-C, represents a characteristic manifestation of metabolic imbalance under high-lipid dietary conditions (Wei et al., 2024; Yang et al., 2023). Betaine supplementation effectively reversed these pathological shifts, with triglyceride levels in betaine-treated groups decreasing below those of the control group. This lipid-normalizing capacity aligns with previous research documenting similar improvements in serum lipid profiles following betaine administration in other aquaculture species, including *A*. *schlegelii* and *Megalobrama amblycephala* (Jin et al., 2021; Adjoumani et al., 2017). The mechanism underlying this effect may involve betaine-mediated enhancement of hepatic phosphatidylcholine synthesis, which promotes VLDL assembly and triglyceride export, as established in mammalian metabolic studies (Buonaiuto et al., 2025). The concurrent elevation of HDL-C, a cardioprotective lipoprotein, further demonstrates the ability of betaine to restore transport balance. These improvements in serum biochemical profiles not only signify recovered metabolic homeostasis but also correlate directly with the transcriptional reprogramming of hepatic lipid metabolism examined in the following section.

The present study reveals a sophisticated transcriptional reprogramming of hepatic lipid metabolism in *M. salmoides* under different dietary regimens. Our findings demonstrate that HF feeding induced a state of metabolic dysregulation, characterized by the significant suppression of genes governing fatty acid oxidation, such as the central transcriptional regulator *pparα*, and its key downstream target *cpt-1*, which mediates the rate-limiting step in mitochondrial fatty acid import. This was accompanied by the downregulation of genes critical for lipolysis, including *lpl*, responsible for hydrolyzing circulating triglycerides, and *hsl*, which mobilizes intracellular lipid stores. This coordinated repression of the catabolic machinery suggests a compromised capacity for lipid utilization, a phenomenon also reported in *Oreochromis niloticus*, *Larimichthys crocea* and *A. schlegelii* fed HF diets, where similar downregulations in *pparα* and *cpt-1* were linked to hepatic lipid accumulation (Jia et al., 2024; Yan et al., 2015; Jin et al., 2021). Dietary betaine supplementation effectively reversed this metabolic disorder through a dual mechanism. It robustly enhanced lipid catabolism by upregulating *pparα*, *cpt-1*, *lpl*, and *hsl*, thereby promoting fatty acid oxidation and lipolysis. Concurrently, it strongly suppressed the lipogenic pathway, reducing *srebp-1* and *fas* expression. This dual action of simultaneously enhancing lipid breakdown and inhibiting its synthesis is a key feature of betaine, as observed in studies on *M. amblycephala* (Adjoumani et al., 2017) and *Siniperca chuatsi* (Li et al., 2024). The coordinated transcriptional shift induced by betaine establishes a metabolic state that prioritizes lipid utilization over storage. These findings align with existing research and provide a clear molecular explanation for the improved lipid homeostasis seen with betaine supplementation. This solidifies betaine’s role as an effective metabolic modulator for mitigating hepatic steatosis in *M. salmoides* under HF dietary stress.

In fish, the inflammatory response constitutes a fundamental component of the innate immune system, serving as a crucial defense mechanism against pathogens and tissue damage (Zou and Secombes, 2016). However, the balance between pro-inflammatory and anti-inflammatory signals is critical for maintaining immune homeostasis and preventing chronic inflammation that can lead to tissue injury and metabolic dysfunction (Zhao et al., 2021). In the present study, HF feeding induced a pronounced pro-inflammatory state in the liver of *M. salmoides*, characterized by significantly upregulated expression of pro-inflammatory cytokines including *tnf-α* and *il-1β*, alongside suppressed expression of anti-inflammatory cytokines such as *tgf-β1* and *il-10*. This inflammatory imbalance mirrors observations in other aquaculture species under HF dietary stress, including *M. amblycephala* and *Oncorhynchus mykiss*, where similar cytokine dysregulation has been documented (Dai et al., 2019; Zhao et al., 2023b). Betaine supplementation effectively restored this inflammatory imbalance, significantly suppressing pro-inflammatory mediators while enhancing the expression of anti-inflammatory cytokines. The anti-inflammatory activity of betaine is known to operate through several key mechanisms: improving sulfur amino acid metabolism to alleviate oxidative stress, directly inhibiting the NF-κB signaling pathway, and suppressing NLRP3 inflammasome activation (Jin et al., 2021; Zhao et al., 2018). Importantly, these anti-inflammatory properties are closely linked to betaine’s roles in redox balance and lipid metabolism, forming an integrated protective network. Since oxidative stress and lipid peroxidation products are established triggers of inflammatory pathways, and inflammation can in turn exacerbate oxidative damage, a self-perpetuating cycle of metabolic deterioration often ensues. By concurrently mitigating oxidative stress, optimizing lipid homeostasis, and resolving inflammation, betaine interrupts this vicious cycle at multiple nodes. Such multi-faceted protection underscores the potential of betaine as a promising functional feed ingredient for comprehensive health management in aquaculture, especially under dietary regimes that provoke metabolic and immune dysregulation. The capacity of betaine to synchronize antioxidant, metabolic, and anti-inflammatory responses represents a valuable strategy for enhancing fish welfare and production efficiency in commercial aquaculture.

## Conclusion

This study demonstrates that dietary betaine supplementation effectively counteracts the adverse effects of a HF diet in largemouth bass through a multi-faceted mechanism. Betaine restored growth performance and feed efficiency, which was associated with its ability to ameliorate HF-induced impairments in digestive function. A key finding is the potent activation of the Nrf2-Keap1 antioxidant pathway, leading to enhanced gene expression and activities of critical antioxidant enzymes, which collectively alleviated hepatic oxidative stress. Concurrently, betaine induced a favorable reprogramming of hepatic lipid metabolism, shifting the balance from lipid storage toward oxidation and catabolism. This transcriptional shift was consistent with the observed improvement in serum lipid profiles. Furthermore, betaine resolved HF-triggered chronic inflammation by rebalancing pro-inflammatory and anti-inflammatory cytokine expression. The coordinated improvement across antioxidant defense, lipid homeostasis, and inflammatory status underscores betaine’s role as a holistic metabolic modulator. Therefore, betaine is a highly promising functional ingredient for aquafeeds, offering a strategic nutritional approach to enhance health and productivity in carnivorous fish species under high-fat dietary regimes.

## Data Availability

The datasets presented in this study can be found in online repositories. The names of the repository/repositories and accession number(s) can be found in the article/supplementary material.
